# Challenges for ticks and tick-borne diseases research in Southeast Asia: Insight from the first international symposium in Cambodia

**DOI:** 10.1371/journal.pntd.0012269

**Published:** 2024-07-10

**Authors:** Sony Yean, Didot Budi Prasetyo, Sebastien Marcombe, Upik Kesumawati Hadi, Abdul Rahman Kazim, Sonthaya Tiawsirisup, Vu Duc Chinh, Keita Matsuno, Van Lun Low, Sarah Bonnet, Nathalie Boulanger, Tommy Tsan-Yuk Lam, Mohammad Yazid Abdad, Vincent Herbreteau, Jean-Marc Chavatte, Samuth Sum, Theary Ren, Anavaj Sakuntabhai, Pierre-Olivier Maquart, Antsa Rakotonirina, Sebastien Boyer

**Affiliations:** 1 Medical and Veterinary Entomology Unit, Institut Pasteur du Cambodge, Phnom Penh, Cambodia; 2 Vector Control Consulting—South East Asia, Vientiane, Lao PDR; 3 School of Veterinary Medicine and Biomedical Sciences, IPB University, Bogor, Indonesia; 4 Department of Medical Microbiology and Parasitology, Faculty of Medicine, Universiti Teknologi MARA, Sungai Buloh Campus, Jalan Hospital, Selangor, Malaysia; 5 Faculty of Veterinary Science, Chulalongkorn University, Bangkok, Thailand; 6 National Institute of Malariology, Parasitology and Entomology, Hanoi, Vietnam; 7 International Institute for Zoonosis Control, Hokkaido University, Sapporo, Japan; 8 Tropical Infectious Diseases Research and Education Centre, Universiti Malaya, Kuala Lumpur, Malaysia; 9 Ecology and Emergence of Arthropod-borne pathogens Unit, Institut Pasteur, Université Paris Cité, CNRS UMR 2000, INRAE USC 1510, Paris, France; 10 University of Strasbourg and French Reference Center Lyme, Strasbourg, France; 11 Centre for Immunology and Infection, Hong Kong; 12 Mahidol Oxford Tropical Medicine Research Unit, Bangkok, Thailand; 13 Institut de Recherche pour le Développement, Phnom Penh, Cambodia; 14 National Public Health Laboratory–National Centre for Infectious Diseases, Ministry of Health, Singapore; 15 Faculty of Veterinary Medicine, Royal University of Agriculture, Phnom Penh, Cambodia; 16 National Animal Health and Production Research Institute, General Directorate of Animal Health and Production, Ministry of Agriculture, Forestry and Fisheries, Phnom Penh, Cambodia; Chengde Medical University, CHINA

## Abstract

**Background:**

Ticks, as critical vectors of a variety of pathogens, pose a significant public health challenge globally. In Southeast Asia (SEA), ticks are responsible for transmitting a diverse array of pathogens affecting humans and animals. The geographical and ecological diversity of SEA provides a unique environment that supports a wide range of tick species, which complicates the management and study of tick-borne diseases (TBDs).

**Methodology/principal findings:**

This article synthesizes findings from the first international symposium on ticks and TBDs in Southeast Asia, held in Phnom Penh on June 22 and 23, 2023. It highlights regional efforts to understand tick ecology and pathogen transmission.

This paper proposes to present a summary of the various presentations given during the symposium following 3 main parts. The first one is devoted to the state of knowledge regarding ticks and TBDs in SEA countries, with presentations from 6 different countries, namely Cambodia, Indonesia, Laos, Malaysia, Thailand, and Vietnam. The second part focuses on the development of new research approaches on tick-borne pathogens (TBPs) and TBDs. The last part is a summary of the round table discussion held on the final day, with the aim of defining the most important challenges and recommendations for researches on TBP and TBD in the SEA region.

**Conclusions/Significance:**

Key topics discussed include advancements in diagnostic tools, such as MALDI-TOF MS and proteomics, and the development of sustainable strategies for tick management and disease prevention. The symposium facilitated the exchange of knowledge and collaborative networks among experts from various disciplines, promoting a unified approach to tackling TBDs in the region. The symposium underscored the need for enhanced surveillance, diagnostics, and inter-regional cooperation to manage the threat of TBDs effectively. Recommendations include the establishment of a regional database for tick identification and the expansion of vector competence studies. These initiatives are crucial for developing targeted interventions and understanding the broader implications of climate change and urbanization on the prevalence of TBDs.

## Author summary

Ticks are arthropods that can transmit pathogens affecting both animals and people. In Southeast Asia, the diversity of tick species and the tick-borne pathogens is particularly high due to the region’s varied climates and ecosystems. Our research, discussed during an international symposium, focuses on understanding these ticks and finding better ways to detect and manage the associated diseases. This symposium highlighted knowledge on ticks and tick-borne pathogens in the different countries, new technologies such as perspectives in research. This work is important because it helps in developing strategies to prevent these diseases, which is crucial for the health of people and animals in Southeast Asia and beyond. This work not only advances scientific knowledge but also supports efforts to improve public health in response to the challenges posed by environmental changes, such as climate and land-use changes.

## 1. Ticks and tick-borne diseases

Ticks are hematophagous arthropods, belonging to the order Ixodida. Around 900 species of ticks are known worldwide, subdivided into the 3 families: Argasidae, Ixodidae, and Nuttalliellidae [1,2]. Ixodidae, commonly known as hard ticks, represent around 80% of the species within the order. The remaining species, excluding a single Nuttalliellidae representative, belong to the family Argasidae, known as soft ticks.

Ticks parasitize various vertebrates, including livestock, wild animals, and humans. This parasitic behavior poses a significant threat to human and animal health, as they serve as vectors for a diverse group of pathogens. In veterinary settings, ticks take on a primary role as vectors, while in the transmission of human pathogens, they rank as the second most important vectors after mosquitoes. The pathogens transmitted by ticks include viruses, bacteria, protozoa, and helminths, whose occurrence depends on various factors including geographical regions [3].

In Southeast Asia (SEA), a range of tick-borne pathogens (TBPs) circulate, including *Anaplasma*, *Borrelia*, *Babesia*, *Coxiella*, *Ehrlichia*, *Rickettsia*, and *Theilaria*. The detection of these pathogens typically employs various methodologies, ranging from basic methods such as blood smear examination, rapid diagnostic kits, and PCR to more sophisticated methods such as sequencing analysis. However, the availability of more advanced diagnostic tools may be limited to renowned universities or research institutes.

To address the growing threat posed by ticks and tick-borne diseases (TBDs) and the disparity in diagnostic capabilities between SEA countries, the establishment of a sustainable network of experts is necessary, facilitating collaborations among multidisciplinary researchers not only from the region but also from the world. The main focus of this network will be to improve the sharing of resources and knowledge to enhance the surveillance, diagnosis, and management of ticks and TBDs. The initial step in accomplishing this goal involves gathering experts from various regions to engage in comprehensive discussions and exchange scientific perspectives through an international symposium, with a particular focus on the Southeast Asian context.

## 2. Brief overview of the symposium

On June 22 and 23, 2023, the first international symposium on ticks and TBDs in SEA was held in Phnom Penh, Cambodia. The symposium was organized by the Institut Pasteur du Cambodge (IPC) in collaboration with the Royal University of Agriculture and the Ministry of Agriculture, Forestry and Fisheries.

The main objective of the symposium was to create a network of experts on ticks and associated pathogens in SEA and promote knowledge transfer and emulation around this emerging topic within the region. Featuring participants from over 10 nationalities, this symposium served as an extensive platform to engage discussion about the current knowledge on ticks and TBDs. It offers optimism in the collective effort to fight against these diseases in the context of climate change and the “One Health” initiative.

This paper provides a summary of the symposium’s presentations and discussion, organized into 3 main parts. The first section addresses the current state of knowledge on ticks and TBDs in SEA countries that were present at this symposium. The contributions made by participants from Cambodia, Indonesia, Laos, Malaysia, Thailand, and Vietnam in their respective presentations collectively enhance the comprehension of a part of the regional knowledge landscape. The second part focuses on the development of new research approaches related to TBPs and TBDs that were presented during the symposium. The last part synthetizes insights from the round table discussion held on the final day, with the aim of identifying the most important challenges and offering recommendations for future research on TBPs and TBDs in the SEA region. The following text synthetized the presentations and the discussions occurring during the symposium.

## 3. State of knowledge in 6 ASEAN countries

Southeast Asia nations are known for their diverse ecosystems, tropical rainforests, and abundant wildlife. They also host a variety of tick species that play an important role in the transmission of TBDs, highlighting the importance of performing studies on ticks in this region. Scientists from 6 SEA countries summarized and presented their work on ticks covering the taxonomical studies and TBPs detection from both tick vectors and animals. Although representatives from other SEA countries attended the symposium, they did not present their work in this session. Therefore, the data from their countries are not included in this current article.

### 3.1. Cambodia

Currently, only 19 tick species from 6 genera are known to be present in Cambodia [4]. The country has witnessed a scarcity of published research on ticks and TBDs, despite their significant importance. In 2022, the medical and veterinary entomology Unit of the Institut Pasteur du Cambodge initiated a cross-sectional study in Cambodia, which enabled the collection and identification of different tick species, along with an exploration of their associations with hosts and environment. The sampling was carried out during the dry season, from November 2022 to April 2023. Tick samples from 4 different ecotypes were collected in 24 of the 25 provinces and cities of Cambodia, excluding the capital, Phnom Penh. The collection involved direct sampling on animals in domestic farms (cattle, pig, and chicken) as well as in veterinary clinics and villages. Additionally, ticks were also collected using flagging and dragging methods in the fringes of forests [5].

In the farm setting, a total of 3,534 ticks were collected from 79 cattle across 8 farms. These belong to 2 species: *Rhipicephalus australis* and *Rh*. *microplus*. Notably, no ticks were found on either chickens or pigs. In the other 3 habitats, we found 3,139 ticks belonging to 13 species. These include 6 new record species for the country: 1 soft tick species (*Carios batuensis*, formerly *Ornithodoros batuensis*) and 5 hard tick species (*Dermacentor filippovea*, *Dermacentor steini*, *Haemaphysalis canestrinii*, *Haemaphysalis hystricis*, and *Haemaphysalis wellingtoni*).

These newly recorded species were found in different locations. While *O*. *batuensis* was collected in bat caves in Stung Treng province, *De*. *filippovea* was sampled on wild pigs in Koh Kong province and *Ha*. *canestrinii* on a dog in Kratie province. The 3 other species were found on the vegetation at the forest fringes in Kampong Chhnang, Kampong Thom, and Stung Treng provinces.

Only a limited number of studies have been conducted to elucidate the circulation of TBPs in livestock in Cambodia. Previous studies have highlighted the circulation of 5 pathogens (*Babesia vogeli*, *Ehrlichia canis*, *Hepatozoon canis*, *Mycoplasma haemocanis*, and *Rickettsia felis*) in dogs across both urban and rural areas in 3 provinces [6,7]. Additionally, several veterinary internship reports from Royal University of Agriculture, Phnom Penh, Cambodia have documented the identification of four pathogen genera (*Anaplasma* spp., *Babesia* spp., *Ehrlichia* spp., and *Theileria* spp.) in both cattle and dogs from 9 provinces of Cambodia. No human cases of TBDs have yet been reported in Cambodia.

No integrated tick control method has been implemented nationwide and the research into integrated tick management strategies remains a challenge in Cambodia. Indeed, tick control measures rely on traditional techniques carried out by individual owners, including manual hand picking, applying salts or tobacco to cattle, and burning grass near cattle shelters, even if some owners opt for commercial insect spray and antiparasitic medicine such as Ivermectin.

All things considered, the circulation of TBDs seems to be confirmed at a larger scale in Cambodia. Significant strengthening of research on ticks and TBD in human and veterinary medicine is needed to better understand their biology and evaluate tick-related risk in this country.

### 3.2. Indonesia

The initial checklist of tick species with their host and distribution in Indonesia was summarized by Anastos [8] and Munaf [9,10]. Later taxonomical works further complete this checklist and recorded a total of 61 species from 7 genera [11–16]. During these works, the most common collection methods used were vegetation flagging and direct host inspection [9,12]. In recent work, the passive tick surveillance approach, involving collaborations with zoo networks and wildlife conservation organizations, is considered as a promising way of extending tick collection to a broader range of vertebrate hosts.

TBPs are present in Indonesia, but the extent of their distribution, particularly in wild vertebrates, is still poorly understood. At least 7 genera of TBPs are known to circulate in animals: *Anaplasma*, *Babesia*, *Borrelia*, *Coxiella*, *Ehrlichia*, *Rickettsia*, and *Theileria*. In domestic animals, *Anaplasma* spp. and *Babesia* spp. have been reported in dogs [16–18] and cattle [19–21]. *Ehrlichia* spp. have been reported in pets such as dogs and cats [16,17,22]. Another hemoprotozoan parasite, *Theileria* spp., has been reported in cattle and buffaloes [20,23]. Recently, *Coxiella burnetii* have also been detected in several internal organs from cattle in West Java [24].

Previous investigation focusing on the detection of TBPs in tick specimens collected from wild animals have revealed the presence of bacteria from the genera *Anaplasma*, *Borrelia*, *Ehrlichia*, and *Rickettsia*. Inspection of exotic pets such as tortoises from 5 locations in and around Jakarta revealed the presence of reptile-associated group (REP) *Borrelia* sp. (closely related to *Borrelia* sp. tAG66M) in the *Amblyomma sparsum* tick species [25]. In another study, *Amblyomma varanense* collected from the monitor lizard *Varanus salvator* were found positive for *Anaplasma* sp. (which phylogenetically related to *Anaplasma marginale* and *Anaplasma bovis*), *Rickettsia* sp. (phylogenetically close to *Candidatus R*. *sepangensis*), and *Borrelia* sp. (closely related to *Borrelia andersonii*, “Candidatus Borrelia tachyglossi”, and *Borrelia turcica*) [15]. In the wild boar (*Sus scrofa*) population, *Anaplasma* sp. and *Ehrlichia* sp. were identified in *Ha*. *hystricis*, while *Borrelia* sp. was detected in *De*. *astrosignatus* and *De*. *steini* from the same host species.

A number of acaricides for tick control in pets and livestock are already available [26] but their management is still lacking and regulations are yet to be enforced. The oldest recorded control method in Indonesia is the direct removal of ticks by hand by owners of small-scale cattle farm [21]. More recently, tick control efforts have focused on the use of commercially available insecticides such as fipronil and the anti-parasitic Ivermectin [27,28].

### 3.3. Laos

The tick fauna of Laos is still largely unknown, but recent progress has been made over the last 10 years. Prior to 2016, studies on ticks in Laos were scare, with only a limited number of articles mentioning the national tick fauna, from 1944 to 2014 [29–40]. An updated checklist of the tick fauna of Laos was published for the first time in 2016 [41]. This checklist, focusing on Khammouane province, identified 15,073 ticks representing 5 genera and at least 11 species. Since then, there have been ongoing efforts making by revisions and updates of species records, descriptions of new species and records from new localities, with articles published regularly [42–46].

There is limited evidence supporting the circulation of TBPs in Laos. Additionally, TBPs are an under-recognized cause of undifferentiated febrile illnesses among adults in Laos. In 2006, a wide diversity of *Rickettsia* was identified for the first time in Laos with the scrub typhus emerging as the most common rickettsiosis identified on patients from Vientiane City and surrounding farming communities of Vientiane Province [47]. Later, the first 2 Lao patients were diagnosed with *Bartonella henselae* endocarditis [48]. Also, a comprehensive study revealed a large panel of ticks’ genus infected with bacteria, carrying potential human disease risks including *Rickettsia* spp., *Borrelia* spp., and *Ehrlichia* spp. [49]. Sequencing results provided evidence for distinct genotypes of bacteria with human disease potential in ticks in Laos [49].

Compared with human TBDs, knowledge on TBPs in animals is even more limited in the country. One study mentioned the presence of zoonotic pathogen in ticks from rural areas [38], while another reported the presence of zoonotic pathogens (*Rickettsia felis*, *R*. *asembonensis*, *Anaplasma* spp., *Leptospira* spp., and *Brucella* spp.) in ticks collected from dogs in the capital Vientiane [50]. These findings suggest a high risk of zoonotic infection for humans in the city. In general, TBDs are significantly underestimated in Laos, and despite extensive research conducted over the past decade, the current state of research remains at an early stage.

### 3.4. Malaysia

Malaysia hosts several species of tick, with a total of 48 species belonging to 7 genera. The most widespread genera are *Haemaphysalis*, *Dermacentor*, and *Rhipicephalus* [30,34,51–62]. These acarine parasites thrive in a variety of habitats, including tropical forests, grasslands, and urban areas, contributing to their widespread distribution across the country. *Rhipicephalus* ticks are known to be common parasites of pets and livestock. For example, *Rh*. *linnaei* and *Rh*. *microplus* parasitize dogs and cattle, respectively. Infestation of these hosts, which are in frequent contact with humans, can expose them to TBPs.

The most studied TBP infecting both animals and humans in Malaysia is *Rickettsia* [2,59]. *Rickettsia conorii* (51/102), *R*. *felis* (23/102), and other tick-borne spotted fever rickettsiae (112/544) have been detected in the “Orang Asli” indigenous population [63,64]. A microbiome survey of ticks collected from domestic animals (dogs, cats, and chickens) identified *Rickettsial* DNA in *Ha*. *bispinosa*, *Ha*. *hystricis*, and *Ha*. *wellingtoni* collected from 2 villages [65]. In addition, a high prevalence of *Rickettsia* was also reported in *De*. *compactus*, a tick of wild boar in Malaysia [66]. While these *Rickettsia* species were identified in tick samples, the capacity of these ticks to transmit the bacteria has not been confirmed. Further studies are therefore needed to investigate the vector competence.

*Anaplasma* studies in Malaysia have primarily focused on investigating the prevalence and molecular characterization of the pathogen in both animals and humans [67,68]. In 2017, a relatively low seroprevalence (7/102) of *Anaplasma phagocytophilum* in human was reported [69]. Additionally, studies have also explored *Anaplasma* infections in wildlife and domestic animals, which may act as reservoirs for the pathogen [70–72]. *Anaplasma* has been reported in a number of ticks in Malaysia, ranging from the forest species such as the reptile-specific *Amblyomma*, to the more cosmopolitan species such as *Rh*. *microplus* and *Rh*. *linnaei* [65,73,74]. Previous studies have demonstrated that the majority of the detected tick-borne *Anaplasma* were zoonotic, such *A*. *phagocytophilum*, *A*. *platys*, and *A*. *marginale* [75,76]. However, there is currently no study confirming the vector competence of these tick species in transmitting *Anaplasma* in Malaysia.

Although not as prevalent as *Rickettsia* and *Anaplasma*, *Borrelia* has also been reported in the population [77]. However, it was not possible to confirm which tick species transmitted the pathogenic spirochete to the affected individuals. Other *Borrelia* species have been documented in several tick species, namely *Borrelia yangtzensis* in *Ixodes granulatus* and *Borrelia* sp. in *Ha*. *hystricis* [78,79]. In summary, much more detailed studies are needed to determine the prevalence of borreliosis in Malaysia.

### 3.5. Thailand

Thailand hosts a great diversity of tick species with 62 species belonging to 8 genera [4]. Studies conducted in the country have focused on the Ixodidae family, including a large number of species of medical and veterinary importance. The most prevalent ticks in Thailand include *Rh*. *sanguineus*, commonly found on dogs, and *Rh*. *microplus* which is predominant on cattle. *Babesia*, *Ehrlichia*, and *Hepatozoon* species are important TBPs in dogs, while *Anaplasma*, *Babesia*, and *Theileria* are important TBPs in cattle [4]. Various tick species have also been found in wildlife and zoos, including *Argas robertsi*, *De*. *auratus*, *Ha*. *lagrangei*, *Ha*. *wellingtoni*, and *Rh*. *microplus*. Nevertheless, their involvement in the transmission of pathogens within wildlife settings remains poorly understood at present.

Thailand is also home to open Zoos located within wildlife sanctuaries, such as the zoo in Chonburi province, featuring a diverse collection of over 300 animal species. And certain wild species are able to move between these 2 zones (zoo and wild areas), and TBPs could potentially be transmitted among wild animals. Given the variety of species and transmission possibilities, the Open Zoo can be an excellent resource for studying tick diversity and their role as vectors of important pathogens.

A specific study conducted in this context identified 4 tick species: *Rh*. *microplus*, *Ha*. *lagrangei*, *Ha*. *wellingtoni*, and *De*. *auratus*. In these ticks, *Anaplasma* spp. exhibited the highest infection rate (247/425), followed by *Babesia* spp. (130/425), and *Theileria* spp. (71/425). Coinfections and tri-infections were found in some collected ticks.

These findings indicate that certain tick species in Thailand could potentially spread TBDs to wild animals in open zoos. This hypothesis is corroborated by the detection of related pathogens in the areas studied. The information obtained could be useful in developing plans for the treatment, prevention, and management of TBDs. However, further research is needed to determine the ability of ticks to transmit these pathogens and to better understand the relationship between pathogens, ticks, and hosts.

### 3.6. Vietnam

As in other SEA countries, the data available on ticks, TBPs, and their distribution in Vietnam are currently limited. The first studies on ticks in Vietnam were carried out in the 1920’s [80–82]. Thanks to various subsequent taxonomic works, a total of 65 tick species belonging to 9 genera have been described in Vietnam using local determination keys [4,83–85]. A survey conducted between 1956 and 1977 in the North and between 2015 and 2016 in the South of the country identifies the distribution of different species within the Ixodidae family, including *Rh*. *microplus*, which appears to be widely distributed [86,87]. Various studies have also identified the host spectra of several tick species [84,86]. Four species were found only from domestic animals: *Amblyomma testudinarum* collected from buffalo; *De*. *auratus* from pigs; *Ix*. *granulatus*, *Rh*. *haemaphysaloides*, and *Rh*. *sanguineus* from dogs; *Rh*. *microplus* from cows, goats, and buffalos. In contrast, the following species were exclusively collected on wild animals: *Aponoma crassipes* and *A*. *gervaisi* parasitizing iguanas and pythons and *Argas* sp. parasitizing bats.

Studies on the prevalence of TBPs in arthropod vectors and vertebrate hosts (including humans) are very rare. The low prevalence of pathogens in different tick species has been highlighted. For example, a study carried out in 2016 revealed only 3 pools of *Rh*. *sanguineus* positive with *Rickettsia* out of 299 tested by PCR [88]. Another study conducted in 1962 also demonstrated the presence of *Piroplasmosis* in cows infested with *Rh*. *microplus* [89]. A recent study on ticks collected on dogs in the North of Vietnam showed that among 302 ticks screened by PCR for the presence of selected TBPs, 3 ticks were tested positive for *H*. *canis*, 1 for *E*. *canis*, and 1 for *B*. *vogeli* [90]. In addition, *Rickettsia* bacteria have been found to infect humans in various regions of Vietnam with high prevalence [91].

Recent scientific investigations conducted over the past decade reveal a pronounced abundance and extensive distribution of tick species. Reports also indicated that TBPs are frequently overlooked, despite their prevalence in both humans and animals. The occurrence of TBDs in humans and domestic animals is currently on the rise in Vietnam. Consequently, there is a pressing need to advance research efforts focused on ticks and TBDs.

## 4. Recent technical advances

The initial presentation session offered valuable insights into the current knowledge, areas of focus, and shared interests regarding tick studies in 6 SEA countries. The presentations continued on the progress made in research, with particular emphasis on advances at the proteomic and molecular levels. These include the development of new techniques for identifying tick vectors and their associated pathogens, the creation of vaccines, and the establishment of tick cell lines. It is important to note that these research advances have not been carried out exclusively by SEA countries, but rather by partners from other countries. This session holds significant importance as it provides a platform to familiarize participants with the research and facilities managed by other research groups.

### 4.1. Use of MALDI-TOF MS and other proteomics methods in epidemiology and risk assessment of tick-borne diseases

Matrix-assisted laser desorption ionization time-of-flight mass spectrometry (MALDI-TOF MS) is a technique that generates mass spectra of unique proteins, species-specific “*fingerprints*” [92–94]. In the early 2000s, this technique was developed for bacterial identification [95]. Since then, MALDI-TOF MS has been used in routine clinical microbiology analyses for the systematic identification of microorganisms [92,94,96].

The use of MALDI-TOF MS in medical and veterinary entomology is becoming increasingly widespread. The introduction of MALDI-TOF MS as an economical, rapid, and highly informative tool for arthropod identification and classification has been developed [97,98]. Currently (June 2023), a total of 90 articles in the PubMed database underline the accuracy of this tool in the identification of arthropods of medical and veterinary importance. Interestingly, this technique appears to be a promising tool for global surveillance of arbovirus vectors.

Previous studies have demonstrated the accuracy of this tool for the identification of tick species using the dissected legs and a half-idiosome of Ixodidae tick [99–101]. Currently, the research group from University of Strasbourg in France hosts a home-made database of reference spectra of around 40 tick species out of approximately 900 (10% of which are medical or veterinary importance). In Cambodia, the team from IPC is also developing a MALDI-TOF MS database for mosquito and tick species. To date, the tick database includes 22 specimens belonging to 3 species.

MALDI-TOF MS has also been used to detect microorganisms in arthropods [102,103], and its accuracy has been improved by the use of artificial intelligence technique [104,105]. Ongoing work at IPC is also being evaluated to improve the accuracy of the MALDI-TOF MS using an artificial intelligence technique for both identifying arthropod species and pathogen detection. Preliminary results obtained with mosquito vectors of the Japanese encephalitis virus are encouraging, with 95% accuracy in distinguishing species. The team is also utilizing the same tools to distinguish between experimentally infected and uninfected *Aedes aegypti* and *Ae*. *albopictus* with dengue and chikungunya virus. This research group also intends to explore the application of this tool for detecting pathogens in ticks.

#### 4.1.1. Identification of pathogens in ticks by nontargeted discovery proteomics

The research group from University of Strasbourg in France tested MALDI-TOF MS for the identification of pathogens directly in ticks, i.e., with *Ix*. *ricinus* infected with *B*. *burgdorferi* s.l., the bacterium responsible of Lyme borreliosis (LB). The finding showed that this proteomic technique was not efficient enough to detect the bacteria, as it showed no differences between infected and non-infected ticks. Therefore, to analyze field-collected ticks, nontargeted (discovery) proteomic was implemented using protein extraction, SDS-PAGE prefractionation, digestion, nanoLiquid-ESI-MS/MS chromatography, and bioinformatics searches. The MS/MS data were obtained in a data-dependent acquisition (DDA) mode and a home-made database containing known TBP was used [106]. From a pool of 5 adult ticks, around 50 pathogenic proteins were successfully identified among 2,400 *Ixodes* proteins. These included proteins associated with *Babesia*, *Rickettsia*, different *Borrelia* species, and *Anaplasma phagocytophilum*.

#### 4.1.2. Identification and detection of pathogens in the vertebrate host by multiplexed proteomics

The host skin is a key interface in vector-borne diseases, as pathogens are co-inoculated with the vector’s saliva in the dermis. Most of them multiply, and some pathogens persist in the skin for months [107]. Using LB as a model, *Borrelia*-infected skin biopsies from mice were used to identify bacterial markers of infection by nontargeted proteomics, then optimized their specific detection and quantification by targeted proteomics [106,108]. Currently, the efficacy of this approach is still being tested in patients with local and disseminated Lyme infections. As ticks can be infected by several pathogens, the ability of *Ix*. *ricinus* to transmit pathogens other than *Borrelia* are being investigated. As targeted proteomics can be multiplexed [109,110], a panel of pathogen proteins corresponding to TBPs in the skin of vertebrate host (first mice, then humans) is also being explored.

In conclusion, proteomic techniques can be used differently in epidemiological studies to measure vector competence. MALDI-TOF MS has proved highly effective in accurately identifying tick species. In the vertebrate host, targeted proteomics could be used to identify markers of infection. However, pathogen detection in ticks is still better by molecular biology than by proteomics.

### 4.2. Strategy for the discovery of emerging tick-borne viruses

Genetic detection methods, such as real-time PCR, conventional PCR, and virome analysis by sequencing, can be useful tools for identifying various viruses in ticks. For example, sequencing technologies have enabled to identify divergent emerging tick-borne viruses responsible for human disease like severe fever with thrombocytopenia [111–117] or the new orthonairovirus, Yezo virus (YEZV) [118,119]. Combining these methods with different specificity and completeness can facilitate the discovery of new viruses. Furthermore, to understand the characteristics of newly discovered viruses, virus culture (i.e., virus isolation) should be an essential approach. To broaden our understanding of virus virulence in ticks, virus isolation methods also need to be improved for tick-borne viruses.

### 4.3. Tick cell research in SEA: Bridging the gap in tick-borne disease control

Tick cell lines have been used as important tools for various in vitro research on ticks, their endosymbionts, and TBDs. This platform, providing a consistent and reproducible system for studying TBPs, facilitates investigations into tick–pathogen interaction at the cellular level. Complementing in vivo research challenges, it proves especially useful in obtaining a large number of live ticks for experiments. In recent decades, the expansion use of tick cell lines has expanded to study various aspects of tick biology, physiology, immunity, microbiome, and pathogen transmission [120], leading to a better understanding of TBDs and facilitating the development of effective control strategies.

Despite the thriving in tick cell line research worldwide, its application in low- and middle-income countries has been limited, probably due to difficulties associated with availability, propagation, and maintenance of tick cell lines. To address these issues, the Tick Cell Biobank (TCB) was established to offer tick cell lines for research and training. The main TCB is located at the University of Liverpool, with 3 additional outposts in Asia, Africa, and South America. The TCB Asia Outpost was established in 2018 at the Tropical Infectious Diseases and Education Centre (TIDREC), Universiti Malaya, Malaysia, to provide tick cell resources and training to researchers in Asia. Currently, this outpost houses a total of 18 hard ticks’ cell lines comprising different strains of *Amblyomma*, *Dermacentor*, *Hyalomma*, *Ixodes*, and *Rhipicephalus* species. This outpost also aims to establish tick cell lines indigenous to SEA, as the cell lines available in the collections originated from the United States of America, Europe, the United Kingdom, and Africa, reinforcing their relevance to the study of endemic ticks and TBDs in this region.

Efforts invested in tick cell line research in SEA have yielded limited results, with only a few studies reported in this region. Lim and colleagues in 2017 [121] initiated primary cell cultures from *Ha*. *bispinosa* embryonic ticks in Malaysia: this tick genus was not represented in existing TCB collections or any other known repository. Unfortunately, this culture collapsed after a while, highlighting the need for intense efforts and attention to isolate cell lines from this genus. Recently, the infection rates and replication kinetics of *Rickettsia raoultii* in cell lines derived from *Rh*. *microplus*, *Rh*. *sanguineus*, and *Ix*. *scapularis* have been characterized [122], but the cell lines used were not originated from this region, underlining the need to generate cell lines from species endemic to SEA.

### 4.4. Tick vaccine

Given the drawbacks of chemical control (development of resistance, environmental hazard, contamination of milk and meat products with drug residues, high cost), there is an urgent need for new approaches that are environmentally sustainable and offer broad protection against current and future TBPs. Molecularly defined vaccines based on protective antigens of ticks are environmentally safe, present reduced risk for resistance acquisition, and hold the potential of protecting against diverse TBDs [123]. To identify tick antigens with a direct effect on vector competence, the team from Ecology and Emergence of Arthropod-borne Pathogens Unit, Institut Pasteur Paris developed a laboratory model of tick transmission of *Bartonella* sp. to identify such candidates in *Ix*. *ricinus* [124,125].

*Ix*. *ricinus* is the most widespread and abundant tick in Europe and is the vector of several TBPs of medical and veterinary importance [126]. As the potential involvement of ticks in the transmission of *B*. *henselae* has been debated for many years, the team first validated the presence of *Bartonella* sp. in several tick populations through epidemiological studies [125,127–129]. The research team then developed an original membrane feeding system to infect ticks from laboratory colonies with a bacterial culture [130,131]. To study the parameters governing tick transmission of bacteria, a functional genomics approach was used to identify genes that are differentially expressed in tick salivary glands (SGs) in response to *B*. *henselae* infection [132].

This study first provided a comprehensive transcriptome analysis of *Ix*. *ricinus* SGs. Secondly, *IrSPI* (*Ix*. *ricinus* Serine Protease Inhibitor) was selected because it showed the highest up-regulation in SGs in response to bacterial infection. Inhibition of *IrSPI* hindered tick feeding and led to a reduction of bacterial load in tick SGs. The next results show that IrSPI harbors the conformational fold typical of Kunitz type I serine protease inhibitors and functionally inhibits elastase [133,134]. Obtained results also show that IrSPI is injected into the host during tick feeding and has no impact on tissue factor pathway-induced coagulation, fibrinolysis, apoptosis, or angiogenesis, but has a significant effect on immune cells as IrSPI affects antigen-presenting macrophages by hampering IL-5 production. In addition, IrSPI represses mitogen-stimulated CD4^+^ 6-cell proliferation, inhibition of T-cell proliferation and leads to marked reductions in the secretion of pro-inflammatory cytokines. The efficacy of IrSPI as a candidate vaccine against tick and bacterial transmission was then evaluated.

Despite validating the safety and immunogenicity of IrSPI in mice and sheep, the results showed that it is not a good candidate vaccine for controlling tick infestation [135]. Indeed, the study provides valuable insights into tick–host interactions and provides information and new protocols that could be exploited to design and evaluate tick vaccines targeting the salivary components involved in the successful feeding of *Ix*. *ricinus* ticks.

## 5. Difficulties, challenges, and priorities

The final session of the symposium features a roundtable discussion involving all the participants, separated into 3 groups. This session aimed to highlight the main difficulties encountered by the different laboratories and governmental entities, and to identify the major challenges and the priorities for the future researches on ticks and TBDs. The outcomes are summarized below in 2 different sections.

### 5.1. Challenges and difficulties on ticks and tick-borne diseases studies in SEA

The 3 working groups identified the lack of talents dedicated to the subjects of ticks and TBD studies as the main problem. This can be explained by a lack of training, research projects, visibility, and awareness of TBDs. The modest perception of veterinary diseases and veterinarians in certain countries, as well as the lack of knowledge of TBDs among humans in comparison to widely known mosquito-borne diseases, could explain this lack of visibility. In addition and directly related to this first point, the lack of capacity-building at universities on these topics could also explain the difficulty of recruiting potential students and researchers. Indeed, a strong lack of interest and attractiveness is observed across multiples countries. In some countries, such as Cambodia, recognizing TBDs and their symptoms are not taught at the University of Science. Moreover, the scarcity of tick taxonomists, coupled with considerable time investment necessary for taxonomic training and the absence of universally applicable tick species determination key, pose a significant problem on developing researches on ticks and TBDs. Consequently, university programs related to vector-borne diseases, one-health and planetary health, especially in related medical-veterinary faculties, should be revised and continuously updated to reflect the current situation of ticks and TBDs in SEA.

Securing funding for tick research is consistently a common difficulty faced by SEA countries. Indeed, all 3 working groups mentioned the problem of research visibility, not only with regard to funders and donors, but also to public decision-makers and governments. Researches in veterinary entomology, particularly regarding ticks and TBDs, are not recognized as a priority in Southeast Asia, despite the significant financial impact of these diseases on livestock farming. Thus, it is important to enhance visibility and awareness of ticks and TBDs among various stakeholders in this region regarding their impacts on both animal and human health and socioeconomic aspects. To achieve this, scientists need to be able to draw on human and animal epidemiological studies, as well as to work more closely with hospitals, animal clinics, and practitioners in contact with potential patients. Forging closer links with local universities and public health authorities also regarded as promising approach.

The discussion groups highlighted that working with just human and veterinary clinical institutions would not be enough to fully understand the ecology of the tick vectors. Multidisciplinary collaborations among research institutes, clinics, conservation groups, animal researchers, and livestock associations (both private and governmental) are necessary to help understand various factors such as transmission efficacy, seasonal and environmental factors leading to the bloom of tick populations. This is especially critical for our colleagues who work heavily in wild areas such as agriculture workers, rangers, etc.

In a more technical aspect, one of the difficulties raised was the absence of an international freely accessible MALDI-TOF MS database for identifying ticks based on their protein spectra, similar to how GenBank serves for species identification through DNA sequences. Establishing such a free-access database will enable species identification at low cost, a factor considered crucial in resource-limited countries. The potential risk for a future privatization of this database poses a threat to progress made in MALDI-TOF MS research in both veterinary and medical entomology. Finally, the absence of dedicated genomic research was emphasized, particularly considering the extensive size of tick genome, thereby increasing the cost of full genome sequencing. Furthermore, the lack of tick survey and collection in SEA also contributes to the scarcity of tick specimens for genomic analyses.

The difficulty of establishing tick colonies in the laboratory has also been recognized as an obstacle in research on TBDs. This difficulty primarily arises challenges in breeding ticks due to: (i) their strict hematophagous behavior requiring prolonged and substantial blood meal; (ii) the variation in host preference among species complicating the use of a one single host for laboratory breeding; and (iii) the complexities involved in setting up an artificial feeding system. Breeding ticks in laboratory could therefore be expensive, requiring substantial quantities of blood that are logistically challenging to obtain in some developing countries.

The initiation of this symposium, the enthusiasm it has generated, and the meetings of scientists working on ticks and their associated diseases is a first step towards establishing a regional network that will have the ambition to last. One of the most difficult challenges will be to maintain this network and find common ground for research.

### 5.2. Research priorities on ticks and TBDs in SEA

As mentioned above, the difficulties and challenges are numerous, and the priorities are more targeted and pragmatic, necessary in the first instance for large-scale research in subsequent years. The working groups have agreed to set a number of approaches to explore priorities in research. The development of the regional tick determination key validated by specialists from each country is considered as one of the initial milestones. In fact, this has been completed partially and a common tick determination key should be released in the years to come.

In parallel to that, the improvement in molecular tick identification has also been mentioned. This includes the advancement of MALDI-TOF MS protein spectra database for tick identification purposes, as well as the need to further develop tick DNA sequence database for gene barcoding. As MALDI-TOF MS was mentioned to be not currently available in many research centers, a completion of gene database for all the tick species in SEA could be better priority for the ticks and TBDs network. In addition, with the development of image recognition through machine learning, the groups raised the possibility of identifying tick adults and nymphs from photos, which would then be analyzed by machine/deep learning and made available through apps on mobile devices.

Once the tick species has been described, the next milestone will be investigating their vector competence. Indeed, the capacity of tick species to infest human or certain animal need to be thoroughly studied to evaluate the risk of pathogen transmission. For this, laboratory-reared tick colonies are needed, as well as access to BSL2 and BSL3 security laboratories depending on the pathogens handled. Access to these biosafety laboratories could be achieved through collaboration, pending the implementation of such laboratories in each country. However, maintaining multi species laboratory-reared tick colonies might be a more challenging task for various partners, as this is very difficult to set up.

With recent changes in land use, climate, and the environment, as well as changes in animal husbandry practices, close contact between man and animal (domestic or wild) is becoming very important. It is imperative to be able to detect the presence of pathogens at an early stage of infection. The use of next-generation sequencing (NGS) by countries in SEA is a key step towards describing the diversity and distribution of TBPs in this region.

With the availability of robust data on ticks, TBPs and TBDs, the next significant step is to develop a digital platform for sharing such data in a secure and efficient way. The tools exist, but there is the question of hosting the data for sharing, and the human resources involved and their responsibility. This platform will serve multiple purposes, from hosting MALDI TOF MS spectra database to hosting distribution map of different tick species, their host, and associated TBPs. Integration with other geospatial data such as land use and climate will allow the scientist to model and predict the risks of TBDs in this region.

Finally, in the framework of pathogen discoveries in ticks and vector competence studies, the development of tick cell lines is important to elucidate the various aspects of pathogen replication and pathogenesis in vitro and to better understand the host–pathogen interactions. It should be noted that almost all existing tick cell lines come from temperate countries, mainly *Ixodes* cells. It is imperative to develop new cell lines from tick species present and predominant in SEA. In addition, the development of tick cell lines offers the possibility of testing specific acaricides directly targeting these cells. For instance, emerging RNAi insecticides are currently developed and will be soon to be used against mosquitoes. These strategies could potentially be adapted and evaluated for their effectiveness against ticks.

## 6. Conclusions

There is a strong desire to advance the research on ticks and TBDs in SEA, and this symposium serves as an opportunity for scientists from the region and around the world to exchange knowledge, research skills, and identify key challenges in this field. Emphasizing data sharing and collaborative efforts in pooling of workspaces, thematic research, and data emerge as a priority for assessing the risks to both human and animal health linked to ticks in the context of One Health and in response to the challenges posed by climate change in SEA.
